# Supplementary material for: Utilization and Regional Differences of In-Patient Services for Peripheral Arterial Disease and Acute Limb Ischemia in Germany: Secondary Analysis of Nationwide DRG Data
Source: J Clin Med. 2022 Apr 11;11(8):2116. doi: 10.3390/jcm11082116 (PMC9025059; doi:10.3390/jcm11082116)
Supplement: Supplementary file 1 [file jcm-11-02116-s001.zip › jcm-1656543-supplementary.pdf]

Supplementary Material:

Table S1: Definition of cases according to ICD-10-GM.

	2005–2014	2015–2018
**PAD Fontaine stage I/IIa**	I70.20	I70.20<br>I70.21
**PAD Fontaine stage IIb**	I70.21	I70.22
**PAD Fontaine stage III**	I70.22	I70.23
**PAD Fontaine stage IV**	I70.23<br>I70.24	I70.24<br>I70.25
**Arterial embolism/thrombosis**	I74.*	I74.*

ICD-10-GM indicates International Statistical Classification of Diseases and Related Health Problems, 10th version, German Modification; PAD, peripheral arterial disease.

Table S2: Case definitions of surgical procedures (A1–A3), endovascular procedures (B), procedures to treat arterial embolism or thrombosis (C), and amputations (D), according to the Operation and Procedure Classification System (OPS) between 2005 and 2008 (source: www.dimdi.de, accessed on 11 March 2022).

(A1) Endarterectomy, patch angioplasty, and other surgical procedures:

OPS Code	Description	Localisation: 5th/6th figure * =
5-381.**	Endarterectomy and patch angioplasty	.33/.34./35 Aorta<br>.5* without .51 Iliac<br>.7* Above knee<br>.8* Below knee
5-382.**	Resection of blood vessels	
5-383.**	Resection and interposition grafting	
5-388.**	Suture of blood vessels	
5-389.**	Surgical occlusion	
5-395.**	Patch angioplasty	
5-396.**	Transposition	
5-397.**	Other reconstructions	

Codes .35/.56/.73/.87 „Vascular prosthesis“ only exist since 2010.

(A2) Bypasses including prostheses:

OPS: 5-393.**	Localisation of proximal anastomosis: 5th/6th figure * =	Localisation of distal anastomosis: 5th/6th figure * =	
Axillary / subclavian artery	.13/.14/.17/.18	–	
Aorta	.3* without .30/.31/.32	–	
Iliac artery	.4* without .48/.49	.33/.35<br>.41	
Femoral artery	.5*	.13/.14/.17/.18<br>.36/.42/.51/.57	
Popliteal artery	.6*	.38<br>.43<br>.52	Not further specified
		.44/.53	Above knee
		.45/.54	Below knee
Crural artery	.7*	.46/.55/.61	
Pedal artery	-	.56/.62	

Codes .34 „aorto-biiliacal“ and .39 „aorto-bipopliteal“ only existed until 2006. Codes .9* „temporary arterio-arterial shunt (intraoperative)“ and .x/.y were excluded.

(A3) Surgical revisions:

OPS: 5-394.*	Description	Localisation:
.0	Surgical treatment of bleeding after vascular operation	Unspecific localisation, no 6th figure intended
.1	Surgical revision of anastomosis	
.2	Surgical revision of vascular implant	
.3	Replacement of vascular implant	
.4	Resection of vascular implant	

(B) Balloon angioplasties and stent implantations:				
**#E1.***	**OPS Code**	**Description**	**Localisation: 6th figure * =**	
#E1.1	8-836.0*	PTA	.*4	Aorta
			.*c	Arteries of lower leg
			.*e	Artificial vessels
			.*q	Other abdominal or pelvic arteries
			.*s	Arteries of thigh
			.*x	Others
#E1.2	8-836.1*<br>8-836.2*	Blade-Angioplasty<br>Laser-Angioplasty	.*4	Aorta
			.*c	Arteries of lower leg
			.*e	Artificial vessels
			.*h	Other abdominal or pelvic arteries
			.*k	Arteries of thigh
			.*x	Others
#E1.3	8-840.0* until .5* 8-841.0* until .5* 8-843.0* until .5* 8-844.0* until .5* 8-845.0* until .1*q	Stent implantation	.*4	Aorta
			.*c	Arteries of lower leg
			.*e	Artificial vessels
			.*q	Other abdominal or pelvic arteries
			.*s	Arteries of thigh
			.*x	Others
#E1.4	8-83c.7*	Intraarterial spasmolysis	No information on localisation	
#E1.5	8-84d	(Percutaneous) transluminal implantation of woven nitinol stents	.*4	Aorta
			.*c	Arteries of lower leg
			.*e	Artificial vessels
			.*q	Other abdominal or pelvic arteries
			.*s	Arteries of thigh
			.*x	Others
#E1.6	8-849.0*<br>8-849.1*	(Percutaneous) transluminal implantation of other uncovered large-bore (>16mm) stents	.*4	Aorta
			.*c	Arteries of lower leg
			.*e	Artificial vessels
			.*q	Other abdominal or pelvic arteries
			.*s	Arteries of thigh
			.*x	Others
8-836.0*: q: Until 2014: .09: „Other abdominal arteries“, from 2015: .09: „Other abdominal or pelvic arteries“, from 2017: .0q s: Until 2016: .0b „Arteries of thigh“, from 2017: .0s. 8-836.1/2*: h: Until 2014: .*9: „Other abdominal arteries“, from 2015: .*9: “Other abdominal or pelvic arteries”, from 2017: .*h. k: Until 2016: .*b “Arteries of thigh”, from 2017: k. 8-83c.7*: Exists only since 2012, previously not available. 8-84d not available before 2018. 8-849.0*/1*: Exists only since 2009, previously not available, q/s similar to stenting.				

(C) Procedures to treat arterial embolism / thrombosis:			
**OPS Code**	**Description**	**Localisation: 5th/6th figure * =**	
5-380.**	Incision, embolectomy	.33/.34./35	Aorta
		.5* without .51	Iliac artery
		.7*	Above knee
		.8*	Below knee

		Including „Vascular prosthesis at given localisation“	
8-836.3*<br>8-836.7*<br>8-836.8*<br>8-836.p*<br>8-836.r*	Atherectomy<br>Selektive Thrombolysis<br>Thrombektomy (excluding rotational thrombectomy)<br>Rotational thrombectomy<br>Kryoplasty	.*4	Aorta
		.*c	Arteries of lower leg
		.*e	Artificial vessels
		.*h	Other abdominal or pelvic arteries
		.*k	Arteries of thigh
		.*x	Others

5-380**: Until 2009, .35/.56/.73/.87 did not exist. Codes cover „vascular prostheses“ and were introduced in 2010.
8-836.**: .*h: until 2014: .*9: „Other abdominal arteries“, from 2016: .*9 „Other abdominal or pelvic arteries“, *h from 2017
*k: until 2016: .*b: „Arteries of thigh“, *k from 2017.

(D) Amputations:

OPS Code	Description
**#A1.1 Major**	
5-864.0-1, 5-864.2	Hemipelvectomy / Hip disarticulation
5-864.3-5	Above knee amputation
5-864.6-7	Knee exarticulation
5-864.8-9, 5-864.a, 5-865.0	Below knee amputation
5-864.x-y	Amputation of extremity, not further specified
**#A1.2 Minor**	
5-865.1-3	Foot amputation
5-865.4-6	Forefoot and midfoot amputation
5-865.7-8	Toe and ray amputation
5-865.x-y	Amputation of foot, not further specified

Figure S1: Regional distribution of treating units in Germany: surgical (vascular or general) units vs. other (angiology, cardiology, internal medicine) units.

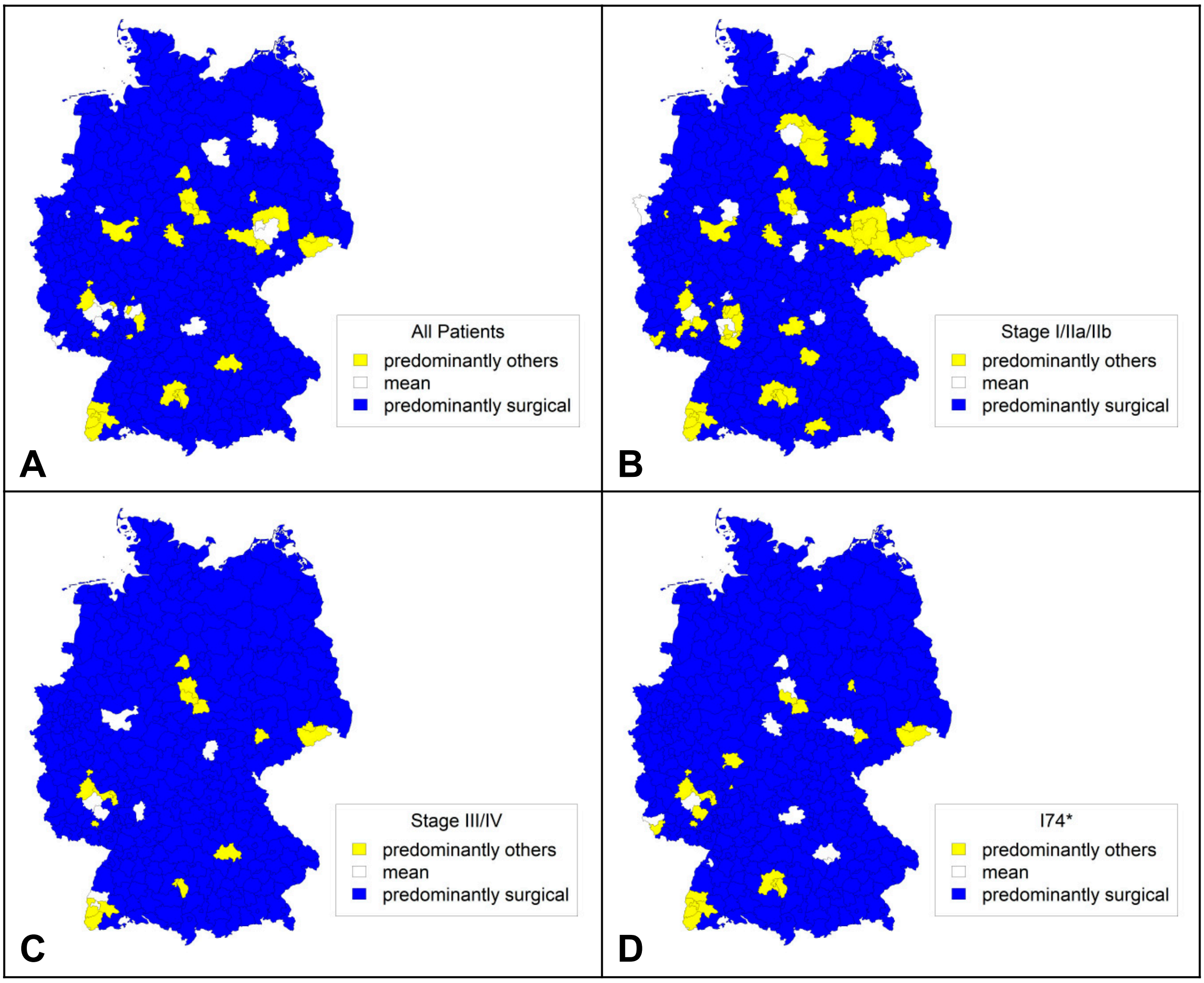

Choropleth maps showing distribution for all patients (**A**), mild peripheral arterial disease (Fontaine stages I, IIa, IIb; (**B**)), critical limb threatening ischemia (Fontaine stages III and IV; (**C**)), and arterial embolism (**D**). *Mean* is defined as the relative difference between features (i.e., cases treated by surgical or other unit) being ≤10% in the respective region. *Predominantly* is defined as the relative difference being >10%.

Figure S2: Regional distribution of treating units in Germany: dedicated vascular (vascular surgery or angiology) units vs. all other (cardiology, internal medicine, general surgery) units.

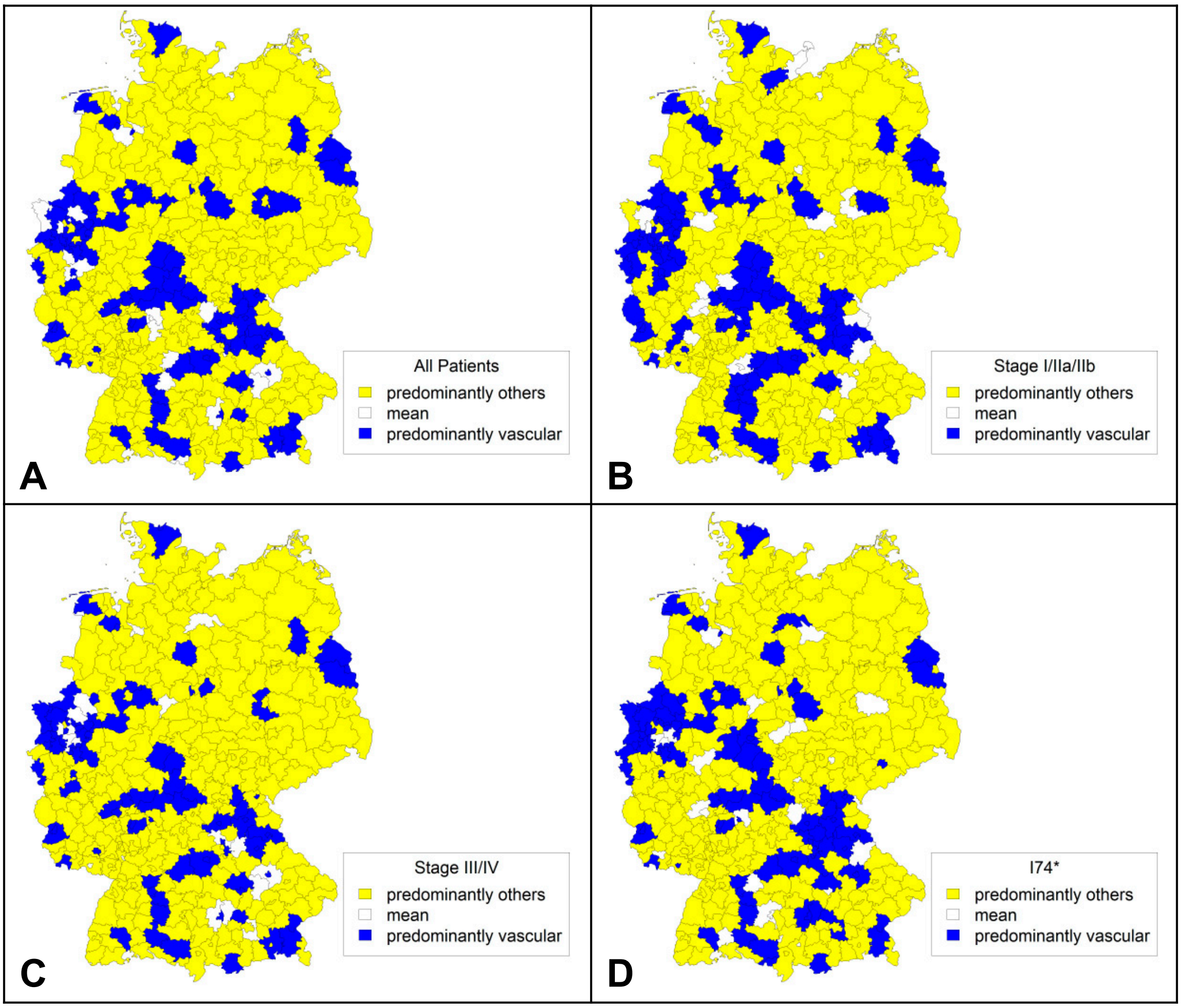

Choropleth maps showing distribution for all patients (**A**), mild peripheral arterial disease (Fontaine stages I, IIa, IIb; (**B**)), critical limb threatening schemia (Fontaine stages III and IV; (**C**)), and arterial embolism (**D**). *Mean* is defined as the relative difference between features (i.e., cases treated by vascular or other unit) being ≤10% in the respective region. *Predominantly* is defined as the relative difference being >10%.
